# Expression of Concern: *PAR-2* promotes cell proliferation, migration and invasion through activating PI3K/AKT signaling pathway in oral squamous cell carcinoma

**DOI:** 10.1042/BSR-20182476_EOC

**Published:** 2021-04-06

**Keywords:** Cell proliferation, Invasion, Migration, Oral squamous cell carcinoma, PAR-2, PI3K/AKT pathway

The Editorial Office has been made aware of potential issues surrounding the scientific validity of this paper, hence has issued an expression of concern to notify readers whilst the Editorial Office investigates.

